# Reconstructive Peri-Implantitis Therapy by Using Bovine Bone Substitute with or without Hyaluronic Acid: A Randomized Clinical Controlled Pilot Study

**DOI:** 10.3390/jfb14030149

**Published:** 2023-03-08

**Authors:** Dragana Rakašević, Miodrag Šćepanović, Iva Mijailović, Tijana Mišić, Bojan Janjić, Ivan Soldatović, Aleksa Marković

**Affiliations:** 1 Department of Oral Surgery, School of Dental Medicine, University of Belgrade, Dr Subotica 4, 11000 Belgrade, Serbia; 2 Department of Prosthodontics, School of Dental Medicine, University of Belgrade, Rankeova 4, 11000 Belgrade, Serbia; 3 Institute for Biostatistics, Faculty of Medicine, University of Belgrade, Dr Subotica 8, 11000 Belgrade, Serbia

**Keywords:** peri-implantitis, reconstructive therapy, intrabody defects, biomaterials, bone substitute, hyaluronic acid

## Abstract

Background: The present pilot study aimed to assess clinical and radiographic efficiencies of bovine bone substitute (BBS) merged with hyaluronic acid (HA) in peri-implantits reconstructive surgery. Methods: Peri-implantitis (diagnosed 6.03 ± 1.61 years of implant loading) bone defects were randomly treated either with BBS plus HA (test group) or BBS alone (control group). Clinical parameters including peri-implant probing depth (PPD), bleeding on probing (BOP), implant stability (ISQ), and radiographic changes in vertical and horizontal marginal bone (MB) levels were assessed at six months postoperatively. New temporary and permanent screw-retained crowns were made at two weeks and three months postoperatively. Data were analyzed using parametric and non-parametric tests. Results: In both groups, 75% of patients and 83% of implants achieved treatment success after six months (no BOP, PPD <5 mm, and no further MB loss). Clinical outcomes improved over time within groups; however, without significant difference between them. ISQ value obtained significant increases in the test compared to the control group at six months postoperatively (*p* < 0.05). The vertical MB gain was significantly greater in the test group compared to the control (*p* < 0.05). Conclusions: Short-term outcomes suggested that BBS merged with HA could improve clinical and radiographic outcomes in peri-implantitis reconstructive therapy.

## 1. Introduction

Modern implant dentistry is struggling with functional complications affecting osseointegrated implants, endangering not only implant stability but subsequently leading to implant loss. The biological complication is known as peri-implantitis, representing the most common and difficult complication caused by dysbiotic dental biofilm and aberrated immunological host response, resulting in non-reversible progressive destruction of supporting tissues and bone resorption [1,2,3]. Clinically, peri-implantitis is characterized as an inflammatory process in the mucosa around the previously osseointegrated implant in function coupled with radiographic marginal bone (MB) loss [4]. The incidence rate of peri-implantitis is currently estimated to be approximately 22% [5], and is expected to increase over the next few years.

Considering the peri-implantitis complexity and disease severity, the therapy is continuing to be a challenge [6]. A variety of therapeutic options has been set in peri-implantitis management aiming to arrest disease progression and further bone loss [7]. The possibility of pathogenic microbes and their products to adhere inside the pits and grooves on rough titanium implant surfaces leads to complex issues in terms of their elimination, especially when a non-surgical peri-implantitis approach is carried out [8,9]. Therefore, surgical therapy is still considered the “gold” standard in peri-implantitis management allowing easier access to the bone defect and „under eye control” intrabony debridement and implant surface decontamination [10]. Open flap debridement and resective surgical peri-implantitis approaches demonstrated the improvement of the implant surface decontamination with a significant reduction in inflammatory signs such as bleeding on probing (BOP) and suppuration (SUP) along with decrease in peri-implant pocket depth (PPD) [11,12,13]. In spite of that, these methods did not achieve complete disease remission [14,15,16,17], resulting in different levels of treatment failure or implant loss (20–60%) in the short-term basis. As a result, the treated implant sites remain meagre in the original supporting bone structure causing the reduction of previously achieved implant stability. Furthermore, engaging with lever force could lead to implant mobility and subsequent implant loss.

The reconstructive surgical approaches of peri-implant bone (PI-B) defects have been described in numerous clinical and animal studies in peri-implantitis management. Depending on the type of peri-implantitis lesion and bone configuration defects [18], various bone graft materials (e.g., autogenous-, allogenic-, xenogenic-, and alloplastic bone) have been utilized alone or combined with non-resorbable and bioresorbable membranes [19,20,21]. Performed with proper implant surface decontamination methods, the bone augmentation resulted in a higher success rate of disease resolution, enhancing clinical outcomes, and MB gain with possible re-osseointegration. It is noteworthy that xenogenic bone or xenograft was one of the most widely used bovine bone substitutes (BBS) in augmentation approaches, mainly due to its well-defined osteoconductive properties and a low resorption rate [22]. The studies revealed greater clinical and radiographic outcomes of BBS compared to both autogenic and alloplastic bone grafts [19,23]. Unfortunately, none of these studies achieved a complete peri-implantitis remission. In contrast, some studies have suggested that BBS should only be considered a filler for bone defects, achieving neither bone formation nor re-osseointegration [24], with poor clinical benefits [25]. The lack of osteogenetic and osteoinductive properties of BBS could be one of the possible explanations for this inadequate achievement, as well as its unlikelihood to obtain bone regeneration equal to the autogenic bone graft.

Consequently, to overcome these issues, different bioactive materials including growth factors (e.g., BMP-2 [26]), platelet-rich fibrin [27], enamel matrix derivative (Emdogain) [28], and hyaluronic acid (HA) [29] were tested alone or combined with BBS in different regenerative surgical procedures to facilitate and increase bone formation and re-osseointegration. Recently, HA as one of the essential regulators of various cell activities (i.e., proliferation, differentiation, adhesion) has been combined with a BBS. HA or hyaluronan, is an anionic, non-sulfated glycosaminoglycan which presents the main natural component of the extracellular matrix. It could be found in numerous tissues and organs including periodontal tissue and alveolar bone as well as in fluids such as saliva, serum, and gingival crevicular fluid [30]. Current literature showed pro-angiogenic effects of HA [31], representing its important role in wound healing acceleration. In vitro studies found that BBS merged with HA could accelerate and improve bone formation by enhancing the angiogenesis and human osteogenic cells viability, migration, and proliferation which implies its important role in the osseo-regenerative procedure [32]. In addition, HA was represented to affect connective and bone tissue reparation [33,34], indirectly influencing a bone formation by retaining osteoinductive growth factors and encouraging angiogenesis and (neo-)vascularization of endothelial cells [35]. HA alone or combined with autogenous bone graft was found to contribute to the improvement of clinical outcomes after surgical periodontal and non-surgical peri-implantitis therapies [29,36].

Even though HA is known as a bio-modification tool for improving bone regeneration, no randomized clinical studies have been conducted to estimate the influence of HA merged with BBS in peri-implantitis surgical therapy. Accordingly, this clinical pilot study aimed to evaluate the short-term clinical and radiographic efficiency of BBS merged with HA in peri-implantitis defects (PI-D) reconstruction by comparing it to BBS without HA, three and six months after peri-implantitis surgery. A null hypothesis was established that there was no difference between BBS merged with HA and BBS without HA by assessing the clinical and radiographic outcomes of peri-implantitis, including BOP/SUP absence (−), PPD reduction, and MB gain.

## 2. Materials and Methods

### 2.1. Study Design and Participants

The current pilot study was designed as randomized controlled clinical trial carried out from November 2021 to July 2022 at the Departments of Oral Surgery and Prosthodontics, Faculty of Dental Medicine, University of Belgrade, Serbia. It was conducted according to the Helsinki Declaration of 1975, revised in 2013, following the CONSORT 2010 statement.

Patients with one or more signs of peri-implantitis (*n* = 21) were assessed for the possible recruitment [4]. Only patients who met all eligible criteria were included for the further peri-implantitis treatment:

1. Minimum 18-year-old;
2. Able to understand the information about the protocol of the study and to sign the inform consent before the treatment;
3. Systematically healthy or with mild or moderate well-controlled systemic conditions or diseases including cardiovascular diseases (i.e., patients with hypertension and arrhythmia);
4. No previous surgical peri-implantits treatment;
5. Able to maintain adequate oral hygiene (O’Leary plaque index score <25%) [37];
6. Presence of at least one implant (bone- or tissue- level) with peri-implantitis defined as: radiography bone loss >2 mm with BOP positive sign (+) at least one side around the implant and PPD >5 mm (mm); In the cases with absence of the previous radiogram, the peri-implantitis was defined in accordance to 2017 World Workshop Consensus (BOP +, PPD ≥6 mm and MB loss ≥3 mm) [3].
7. Patients who did not receive antibiotic therapy within last 2 months;
8. Non-smokers or light smokers (<10 cigarettes/day).

However, patients were excluded from the study in the presence of any of the predetermined criteria: (1) Implant mobility, (2) Radiographic records showing ≥ two-third of bone loss (sever peri-implantitis) with or without implant mobility, (3) Implant malposition (4) Untreated periodontal disease, (5) Systemic conditions and diseases, such as diabetes mellitus, leukemia, musculoskeletal diseases, and disorders, (6) Patients on medications known to modified bone metabolism or could influence would healing including high-dosed antiresorptive drug use (i.e., bisphosphonate), (7) Earlier head and neck radiation therapy, (8) Pregnant and lactation, (9) Heavy smoker (≥10 cigarettes/day).

**Figure 1:**
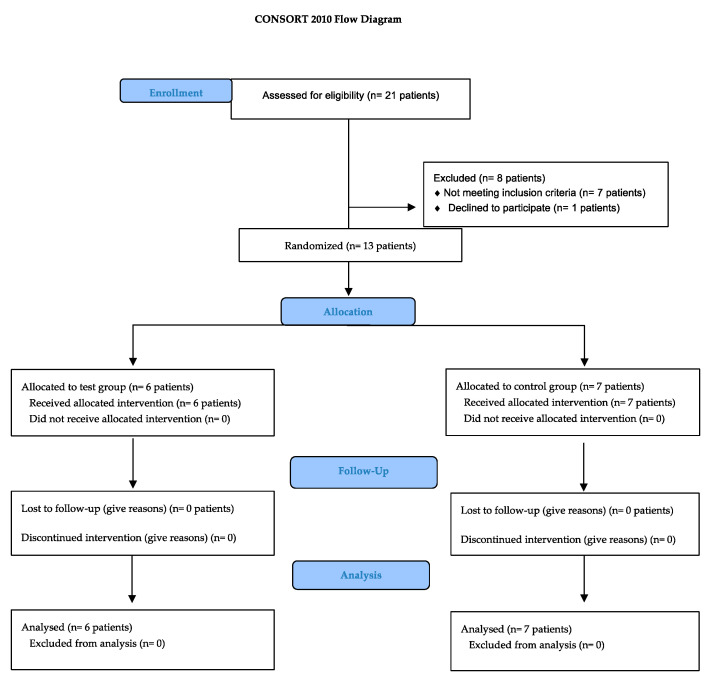
Consort Flow Chart.

Once the patients had met the eligibility criteria (CONSORT flow chart, Figure 1), they received the study protocol divided into four-time (T) points and were asked to sign an informed consent.

The study was previously approved by a local Ethical Committee of the School of Dental Medicine, University of Belgrade, Serbia (numb. 36/25).

### 2.2. Study Protocol

#### 2.2.1. Pre-Surgical Treatment (T0)

**Figure 2:**
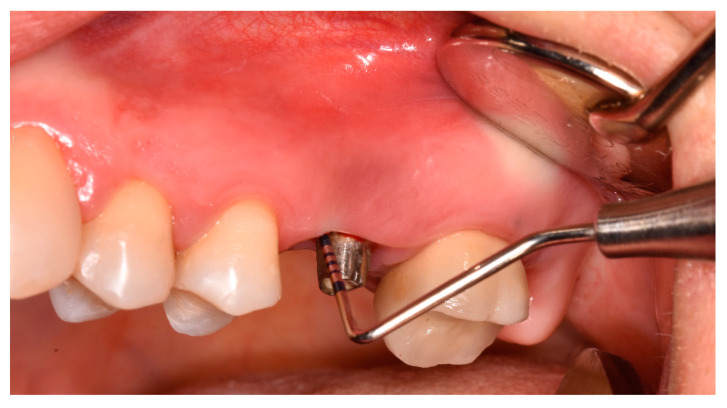
Clinical parameters measured after implant crown removal.

In all patients, prosthetic restauration (implant-retained crown) was removed two weeks before the surgery and replaced with the healing abutment (Figure 2). In the same visit, the impression for the temporary screw-retained crown was taken. Patients received a temporary crown after two weeks postoperatively.

Subsequently, each patient underwent a full-mouth scaling and polishing by means of an ultrasonic device and rubber cap with abrasive paste accompanied by proper oral hygiene instructions, aiming to control and reduce inflammation.

Additionally, non-surgical peri-implantitis therapy was carried out by means of a Gracey titanium curettes (Spain). Antibiotics and antiseptics were not administrated in this phasis.

#### 2.2.2. Surgical Regenerative Approaches (T1) with Patients’ Randomization

**Figure 3:**
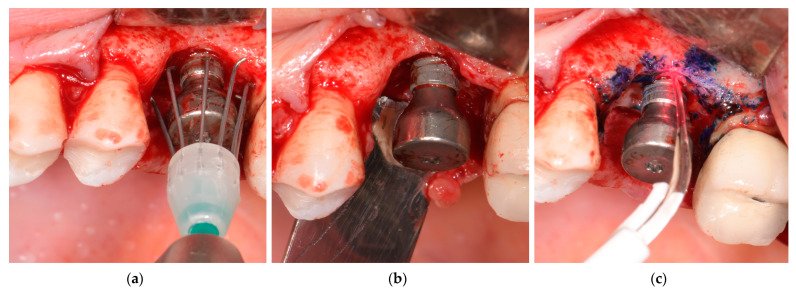
Reconstructive surgical procedure of peri-implantitis: (**a**) Mechanical debridement and implant surface decontamination by means of titanium brush; (**b**) PI-B defect after mechanical debridement; (**c**) Adjunctive implant surface decontamination by photodynamic therapy (PDT).

The established surgical procedure was conducted on all patients by two experienced surgeons (A.M, D.R). Briefly, after local anesthesia (4% articaine with epinephrine, 1:100,000), following intrasulcular incision and flap evaluation, granulation tissue was removed and the implant surface was decontaminated by means of titanium curettes, titanium brushes (R-Brush, Neobiotech, Seoul, Republic of Korea) at 400 rpm under irrigation, and photodynamic therapy (PDT) (HELBO, Photodynamic Systems GmbH, Wels, Austria), earlier described by Rakasevic et al. [6] (Figure 3a–c). In the case of supracrestal exposed treads, implantoplasty was conducted. Following implant surface decontamination, the implant stability (ISQ) was, respectively, measured.

**Figure 4:**
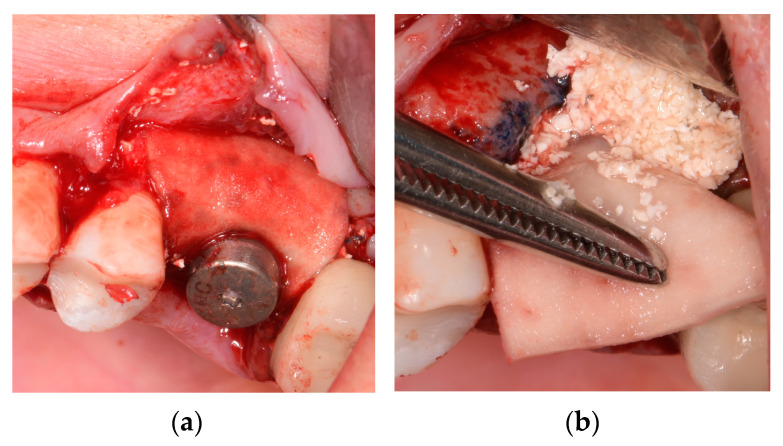
Peri-implant bone defect reconstruction by (**a**) bovine bone substitute (BBS) with hyaluronic acid (HA) covered by (**b**) porcine dermal collagen matrix (PDCM).

Using Microsoft Excel^®^-generated randomization lists, examiners not involved in the surgical procedure and clinical examination prepared sealed envelopes for patients’ allocation. A randomization envelope was opened by the surgical assistant immediately before peri-implant bone (PI-B) defects augmentation, assigning patients to either test (TG) or control (CG) groups. In the TG, PI-B defects were reconstructed by BBS with HA (Cerabone^®^ plus, Botiss Biomaterials GmbH, Berlin, Germany) (Figure 4a), while in the CG, BBS without HA (Cerabone^®^, Botiss Biomaterials, GmbH, Berlin, Germany) was applied. Subsequently, the second ISQ was measured and recorded.

As a concurrent procedure, to affect soft tissue keratinized mucosa (KM) and mucosal thickness (MT) changes, porcine dermal collagen matrix (PDCM) (Mucoderm ^®^, Botiss Biomaterials GmbH, Berlin, Germany) was applied to cover BBS (Figure 4b). PDCM was previously measured and trimmed to completely cover all bone defects in vertical and horizontal dimensions, extending a minimum of 2 mm mesiodistally and apically, ensuring adequate blood supply. Following the manufacturer’s instruction, PDCM was subsequently rehydrated in saline solution (15 min). In addition to fixing the matrix, a healing former was placed above. The flap was sutured coronally with 5–0 absorbable suture (AssuCryl Lactin, Pully-Lausanne, Switzerland) to complete cover the matrix.

#### 2.2.3. Postoperative Instruction, Re-Calls, and Prosthetic Rehabilitation

Postoperatively, antibiotics (Amoxicillin: 500 mg or in case of penicillin allergy Clindamycin: 600 mg, 3 times/day for 5 days) and chlorhexidine solution (0.12%, twice daily for 14 days) were administrated. Patients were advised not to brush operated region within 3 weeks. Surgical sutures were removed 14 days after surgery. Professional dental hygiene around the treated area was carried out every two weeks along with outcomes assessment within the first three months.

Two weeks postoperatively, the provisional previously designed and fabricated crown was replaced, allowing emergency profile formation. Finally, at three months postoperatively (T2), the new customized screw-retained crown was delivered to patients.

### 2.3. Data Collection and Intra-Examiner Calibration

Assessed and collected data comprised clinical, intra-operative, and radiographic examination carried out in four time points including preoperatively (T0), intraoperatively (T1), three (T2), and six months (T3) postoperatively. All clinical examinations were carried out by one single-blinded calibrated clinician (I.M), and radiography assessment was conducted by other single-blinded calibrated clinician (T.M). Both examiners were not included in the surgical intervention, therefore, were masked in patient’s group allocation and treatment. Moreover, patients remained masked throughout all study periods.

To establish intra-rater reliability, the examiners repeated the first 4 measurements of PPD (clinical variable) and 10 measurements of MB level (radiographical variable) after a week in random order. The intra-examiner reproducibility resulted in intra-class correlation coefficients of 0.94 (CI 95% 0.90 to 0.96, standard error 0.12) for PPD and 0.90 (CI 95% 0.89 to 0.95, standard error (SE) 0.19) for MB level.

#### 2.3.1. Clinical Examination

As a part of clinical examination, following clinical parameters were obtained at T0, T2, and T3 time points using a graduated probe (PCPUNC 15, Hu-Friedy, Chicago, IL, USA), performing a force of 0.25 N:

1. Peri-implant probing depth (PPD) and clinical attachment level (CAL) were measured at the six sites (bucco-mesial, bucco-medial, bucco-distal, oro-mesial, oro-medial, and oro-distal) and recorded in millimeters (mm). PPD was determined as a distance from the mucosal margin (MM) to the depth of the probable peri-implant pocket, while CAL was obtained as distance from implant shoulder to the bottom of peri-implant pocket;
2. Modified bleeding index (mBlI) and plaque index (PI) were scored from 0 to 3 according recorded at four points [37,38];
3. Suppuration (SUP) on probing assessed dichotomously (+/−) after the probing;
4. Keratinized mucosa width (KMW) was measured at the middle of the inserted implant as the distance between the free MM to a mucogingival junction (MGJ).
5. Mucosal thickness (MT) was assessed at middle aspect of the examined implant, 3 mm from the top of the MM, by using endodontic needle (K-file, ISO 25) perpendicularly, under the local anesthesia (LA).
6. Peri-implant osseous defects were estimated during the surgical procedure, following granulation tissue removal and implant surface mechanical decontamination. The defect morphology was classified into categories previously described by Schwarz et al. [18];
7. Healing index (HI) was estimated within one month postoperatively, scored from 1 to 5 according to Landry et al. [39];
8. Periodontitis history presence was assessed as dichotomously (yes/no);
9. Visual analogue scale (VAS) had obtained patient morbidity and discomfort during surgical intervention and within four weeks postoperatively, scored 1 to 10. Cut-off points for both pain and difficulty were ≤3.4 (none or mild), 3.5–7.4 (moderate), and ≥7.5 (severe).

#### 2.3.2. Radiographic Examination

Radiographic examinations assessed changes in MB level around implant at vertical sites (mesial, distal, oral, and vestibular) and horizontal side (mesial and distal) using cone-beam computed tomography (CBCT) at T0 (before any treatment procedure) and T3 time points (6 months after the treatment). CBCT images were obtained using Scanora 3D (Soredex^®^, Tuusula, Finland) using uniform exposure settings of Small FOV (60 × 60 mm), 6.3.m.A, 6.1 s, 90 kVp, and 0.2 mm voxel size. Linear measurements were performed by OnDemand3D^®^ software (Cybermed Inc. Daejeon, Republic of Korea).

By using a „curve tool” in the software’s axial window, a panoramic curve was set to cut the implant across its mesiodistal diameter, including its center. MB levels were evaluated mesially and distally at the panoramic window of the software. A cross-sectional slice was appointed through the middle of the implant diameter to assess MB levels at the vestibular and oral implant sides. The vertical MB (V-MB) level was estimated from the implant shoulder representing a reference point to the first visible bone-to-implant contact. The horizontal MB (H-MB) level was assessed from the implant shoulder to the bone crest. Measurements were expressed in mm using a “ruler tool” of the software.

To calculate bone gained six months postoperatively, the value recorded at six months was subtracted from the baseline value (Δ BG). CDs with CBCT recordings were labelled with random numbers to mask the type of intervention and the time of measurement.

#### 2.3.3. Implant Stability (ISQ) Examination

Implant stability was measured by means of Penguin^®^ (Integration Diagnostics; Sweden) after prosthetic crown and suprastructure removal (T0); during intra-operative procedure (T1) following implant surface decontamination and intrabony defect augmentation; as well as at one month; three (T2); and six (T3) months postoperatively.

#### 2.3.4. Success of Augmentation Therapy

Peri-implant treatment success at six months was considered if there were BOP/SUP’’−‘’, along with PPD ≤5 mm and no further radiographic MB loss.

### 2.4. Statistical Analysis

Statistical analysis was performed using SPSS 20.0 (IBM Corp. Released 2011. IBM SPSS Statistics for Windows, Version 20.0., IBM Corp., Armonk, NY, USA). Results were presented as count (%), means ± SD or median (25th–75th Percentile) depending on data type and distribution. Data were analyzed using parametric (*t* test) and nonparametric (Chi-square, Mann–Whitney U test) tests. All *p* values < 0.05 were considered significant.

## 3. Results

### 3.1. Demographic Data and Implants-Based Characteristics

Out of 21 patients with peri-implantitis signs, the pilot study included 13 patients (mean age 46.85 ± 9.96). In total, 19 peri-implantitis with moderate (63%) and advanced disease severity and were diagnosed after 6.03 ± 1.61 years of implant loading (Table 1) and underwent treatment procedures. No adverse or side effects including the implant lost and allergy were reported at six months of follow-up. Two patients displayed flap dehiscence at two weeks postoperatively with slight erythema and matrix exposure, still, without suppuration and patients’ discomfort. However, the healing was uneventful for other patients. Mostly peri-implantits occurred in the maxilla (74%) with sandblasted, large grit, acid-etched, SLA (53%) and blasted-etched (26%) implant surface. Apart from one tissue-level implant, all implants were bone-level with moderate roughness of implant surfaces. Remained cement was found in around 21%.

### 3.2. Clinical Outcomes Assessment

**Figure 5:**
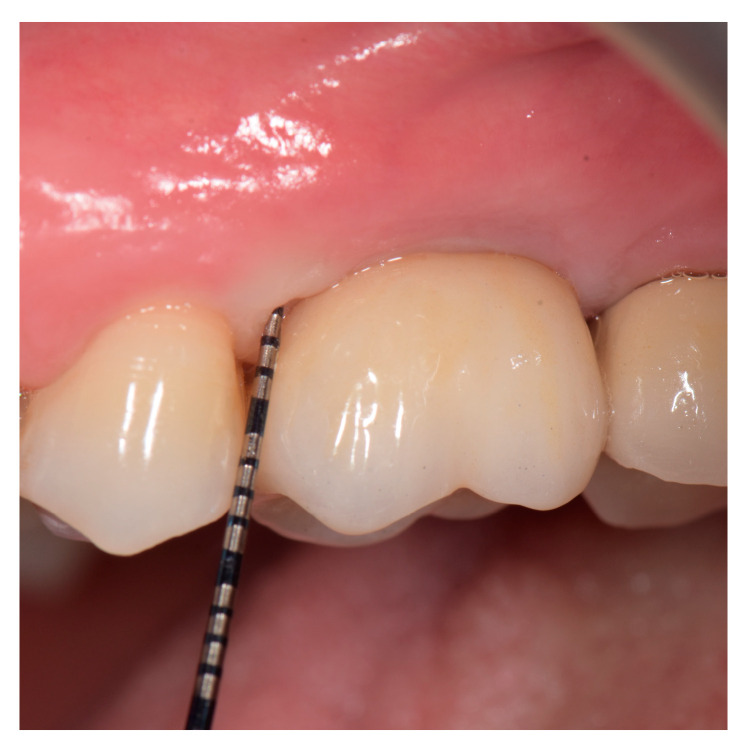
Clinical outcome six months after reconstructive surgical peri-implantitis therapy with novel prosthetic restoration (screw-retained crown).

Treatment success was achieved in 75% of patients and 83% of implants in both groups (BOP/SUP’–, PPD < 5 mm, and no further bone loss). Clinical outcomes improved over time within both groups (Table 2) (Figure 5). In both groups, almost all implants had high mBlI scores prior to treatment, without statistically significant differences. Interestingly, the test group showed a complete reduction in BOP (mBlI: 0) in comparison with the control group (mBlI: 0.17 ± 0.39), six months postoperatively.

**Figure 6:**
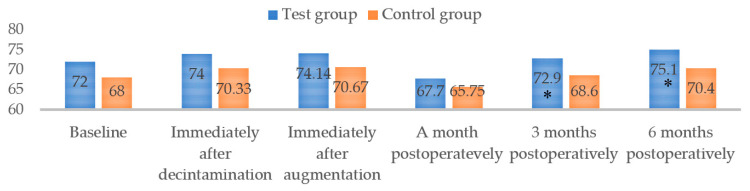
Implant stability (ISQ) values measured at different time points; * Statistically significant difference between the groups by *t*-test (*p* < 0.05).

However, no statistically significant difference between groups was obtained. In terms of postoperative discomfort, both groups’ VAS scores for surgical procedure difficulty and pain severity during surgery were displayed as moderate (VAS = 6.12 and 5.35, respectively). However, the postoperative VAS score (within 4 weeks of follow-up) was recorded as zero in both groups, implying that postoperative pain was not present. Even though baseline ISQ scores were similar between groups (*p* = 0.271), ISQ scores increased after mechanical debridement and bone augmentation, but without significant differences between groups (*p* > 0.05). Interestingly, in both groups, the ISQ values decreased one month postoperatively, and then gradually increased after three and six months (Figure 6). Results showed statistically greater ISQ values in the TG, compared to the CG, at three and six months (*p* = 0.009 and 0.032, respectively).

### 3.3. Radiographic Outcomes Assessment

**Figure 7:**
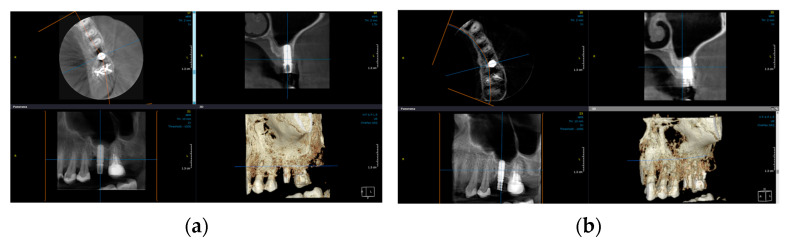
Radiographic imagines representing the marginal bone (MB) changes measured at baseline (**a**) and six months after surgery (**b**).

Considering radiographic parameters, the radiographic analysis demonstrated significant bone gain (Δ BG) within both groups six months after the surgery (*p* < 0.05) (Figure 7a,b). The results showed statistically greater Δ BG in terms of vertical dimension at mesial, distal, and oral sites in the test group compared to the control (*p* < 0.05). Regarding other estimated parameters of radiography, no difference was observed between groups (Table 3).

## 4. Discussion

This study evaluated the efficacy of peri-implant defects reconstruction using either a BBS combined with or without HA at six-month follow-ups in surgical peri-implantitis management. Considering the pilot design of the study with the limited patient number, the short-term results displayed clinical and radiographic outcome improvement within both groups; however, without significant clinical differences between them. Radiographic analysis revealed statistically greater bone gain (Δ BG) in the test group compared to the control one six months after surgery. Interestingly, the implant stability value showed a changeable trend, demonstrating a statistically significantly higher score at three and six months postoperatively in the test compared to the control group (*p* = 0.009 and 0.032, respectively). Accordingly, the previously set null hypothesis was rejected since the test group (BBS merged with HA merged) demonstrated an improvement in outcomes compared to the control group (BBS without HA). To the best of our knowledge, this is the first study in which BBS with/without HA was used simultaneously with porcine dermal collagen matrix for peri-implantitis hard and soft tissue reconstruction. Therefore, the overall results were not attainable to compare to other previously published results of the studies.

The reconstructive peri-implantitis approach has been suggested as a feasible concept in the presence of PI-B defects [18], aiming to achieve bone regeneration, re-osseointegration, and, respectively, limit peri-implant mucosa recession occurrence [40]. Considering various PI-B defect types as one of the determining factors influencing treatment outcomes [18], an MB gain of 2 mm with 2.8 mm of PD reduction was obtained by performing this approach [41], which is in accordance with our gained results. However, according to the study of Schwarz et al. [18], as opposed to our results, Class Ib and Ic defects could make a less favorable biological environment for the BBS, resulting in a lower clinical outcome success after six months of treatment. In the present study, almost 57% and 22% of the PI-B defects had class Ib and Ic defects, representing the greater reduction of clinical outcomes including PPD, CAL, and BOP in both tested groups compared to baseline measurements. Similarly, the study by Roccuzzo et al. demonstrated successful peri-implantitis resolution with the improvement of clinical outcomes such as PPD and BOP, reconstructing Ib (*n* = 36%) and Ic (*n* = 20%) PI-B defects using 10% collagen-deproteinized BBS [42]. The composition properties of the graft materials used in all the above-mentioned studies could explain the heterogeneity in obtained results.

The choice of bone graft material for PI-B defect reconstruction could be essential in peri-implantitis treatment. Ideally, the graft material should meet specific requests and consist of biological cells which could trigger osteoblast migration and differentiation, leading to the formation of new mineralized tissues, while showing the lowest biodegradability. Throughout the numerous studies, BBS (xenograft) was commonly utilized as a biomaterial asserting better clinical and radiographic outcomes when compared to open flap debridement, autogenous or alloplastic bone graft [7,23]. In spite of that, total successful peri-implantitis resolution by means of BBS has been seldom documented. A possible explanation could be the fact that BBS does not have equal regenerative potential as autogenous grafts, thus behaving more like a scaffold. Hence, to achieve the additional osteoinductive potential of BBS, it has been combined with various bioactive materials including HA. HA or hyaluronan showed an essential role in biological processes not only for wound healing but also by serving as an osteoinductive growth factor retainer, thereby promoting bone regeneration by stimulating osteogenetic cell differentiation, influencing angiogenesis and neovascularization, and consequently increasing the process of osteogenesis [35]. Recently, systematic review and meta-analysis as well as an experimental study suggested a moderate role of HA in periodontal tissue regeneration following periodontal surgery [31,33], probably due to its fluid consistency which may lead to the collapse of the mucoperiosteal flap. Therefore, combining HA with BBS might be a promising treatment option. In our study, although there were no significant differences, a BBS merged with HA displayed slightly better outcomes in terms of clinical and radiographic parameters, especially regarding BOP absence (which represents one of the important inflammation signs) and MB gain, compared to BBS without HA. This might be due to HA’s ability to act as an anti-inflammatory agent by arresting pro-inflammatory cells production, prompting a major reduction in inflammation and BOP, as well. As a matter of fact, HA’s potential role to up-regulate the CD44 marker [43] results in the stimulation of wound healing and osteogenetic cells migration, which could explain why bone gain and implant stability increased in our study group where HA was present. Similar to present outcomes, the recent clinical study showed PPD and CAL decreases (4.54 ± 1.65 mm, and 3.65 ± 1.67 mm) at 6 months when BBS was combined with HA gel following periodontitis regenerative surgery [44]. Accordingly, it could be assumed that HA in the presence of BBS increases the biological activity of human osteogenic cells, leading to their higher migration and proliferation. Subsequently, this could enhance and facilitate bone regeneration [32].

In addition to PI-B defects configuration, implant surface and its decontamination methods [7,45], along with prosthetic reconstruction, play a critical role in peri-implantitis successful resolution. The possibility of re-osteointegration on previously contaminated surfaces was recently demonstrated [46]. In the present study, for implant surface decontamination, titanium brushes and PDT were utilized. Interestingly, both methods do not alter the implant surface [47,48], which accomplishes PPD reduction, BOP decrease, and MB gain [6,48,49,50], thus creating supportive conditions for re-osseointegration. In line with our results, a recent study noted approximately 3 mm of PPD reduction and 2.4 mm of MB gain at six months postoperatively by using titanium brushes for implant surface decontamination following the reconstructive PI-B defect procedure [49]. Nonetheless, the incomplete reduction of BOP (20%) in the study above could be explained by the inability of the mechanical method alone to eliminate pathogens and their products from the different implant surfaces. Hence, adjunctive methods, such as PDT, should be used in addition to mechanical methods following reconstructive peri-implantitis therapy, since it was demonstrated that PDT combined with BBS can achieve partial re-osseointegration [24]. Consequently, an almost complete reduction in mBlI and PI scores in both groups of our study could be affected by the PDT application. Nevertheless, the successful resolution of BOP in the test group may be influenced by the additional anti-inflammatory and anti-bacterial properties of HA. Furthermore, it could be speculated that pre-surgery prosthetic restauration removal in the present study potentially provided adequate conditions for implant surface decontamination and bone regeneration. Hence, old prosthetic restauration (implant-retained crown) was removed before the surgery and new temporary and permanent screw-retained crowns were designed thereafter to maintain the stability of the results achieved. Long-term follow-ups are required hereby to confirm this statement.

The use of resorbable and non-resorbable membranes in reconstructive peri-implantitis treatment is still being debated. Considering the finding that a resorbable collagen membrane (CM) did not enhance the results of peri-implantitis therapy as well as the possibility of complications including its exposure [51], the CM was not applied in this study. However, deliberating keratinized mucosa’s importance in peri-implantitis occurrence, PDCM was inserted concurrently following PI-B defect reconstruction with the rationale of obtaining the minimal amount of KM necessary for effective sealing [42]. In addition, since the PDCM could be assumed as a CTG substitute, the present study aimed to diminish patient morbidity and shorten the further surgical procedure, trying to act on keratinized mucosa changes and arresting in this way following mucosal recession. Accordingly, a six-month result obtained minimal rises in terms of KMW and MT by applying PDCM. Interestingly, a higher KMW achieved in the test compared to the control group (0.9 ± 1.9 mm vs. 0. 42 ± 0.96 mm) could be attributed to HA’s influence presented in the bone graft. Moreover, another possible explanation for enhancement in keratinized mucosa could be due to novel prosthetic restoration (screw-retained). This suggested protocol might maintain not only the outcome’s stability but also could influence the soft tissue changes and better healing results. Nevertheless, these results should be interpreted with caution considering the small sample size, group allocation, and follow-up periods evaluated in the study. Therefore, a greater number of patients and long-term results are necessary.

## 5. Conclusions

Despite the limitations of the pilot study, BBS with HA demonstrated that it could improve clinical outcomes and prompt MB gain. In terms of peri-implantitis reconstruction, the proposed surgical approach of using BBS merged with HA might be considered effective.

## Figures and Tables

**Table 1:** Demographic and implants characteristics parameters.

		Test Group	Control Group	Total	*p*-Value
Sex, n (%)	Female			8(62)	0.633
History of treated periodontitis, n (%)	Yes			3 (16)	0.523
Periodontitis	Grade II, Stage B			5 (38)	0.634
	Grade III, Stage B			3 (24)	
	Others			5 (38)	
Smokers (<10 cigarettes), n (%)		2 (33)	2 (28)	5 (38)	0.326
Location, n (%)	Maxilla	5 (71.4)	9 (75)	14 (74)	0.999
	Mandible	2 (28.6)	3 (25)	5 (26)	
Implant system, n (%)	Straumann	2 (29)	7 (58)	9 (47.4)	0.425
	Bredent	3 (44)	2 (17)	5 (26.3)	
	Other	2 (29)	5 (25)	5 (26.3)	
Type of restoration, n (%)	Cement-retained, single crown	6 (86)	6 (50)	12 (63.2)	0.173
	Screw-retained, single crown	1 (14)	6 (50)	7 (31)	
Peri-implant osseous defects, n (%)	Class 1 b	4 (58)	7 (58)	11 (57)	0.642
	Class 1 c	1 (14)	3 (25)	4 (22)	
	Class 1 e	1 (14)	2 (17)	3 (16)	
	Class 2 b	1 (14)	0 (0)	1 (5)	

Abbreviations: TG—test group, CG—control group, n—number presented in percentages (%).

**Table 2:** Clinical outcomes measured baseline, three, and six months after the surgery.

	Follow-Up Periods	Test Group	Control Group	*p*-Value
PPD, SD ± mean	Baseline	5.38 ± 1.06	5.10 ± 0.92	0.576
	3 months	2.56 ± 0.78	2.71 ± 0.38	0.573
	6 months	2.34 ± 0.4	2.51 ± 0.39	0.360
	Δ 6 m	3.02 ± 0.94	2.6 ± 0.88	0.333
CAL, SD ± mean	Baseline	4 ± 1.47	3.13 ± 1.49	0.179
	3 months	1.31 ± 0.62	1.83 ± 0.78	0.153
	6 months	2.34 ± 0.1.67	2.5 ± 1.92	0.432
	Δ 6 m	1.64 ±1.09	1.21 ± 0.84	0.341
MT, SD ± mean	Baseline	0.65 ± 0.16	0.90 ± 0.39	0.070
	3 months	1. 51± 0.37	1.45 ± 0.41	0.737
	6 months	1.54 ± 0.59	1.56 ± 0.40	0.921
	Δ 6 m	0.9 ± 0.47	0. 6 ± 0.54	0.25
KMW, SD ± mean	Baseline	2.36 ± 1.97	2.57 ± 2.02	0.578
	3 months	2.93 ±1.17	2.86 ± 1.92	0.318
	6 months	3.26 ± 0.67	2.83 ± 2.01	0.131
	Δ 6 m	0.9 ± 1.9	0. 42 ± 0.96	0.47
mBlI score, n	Baseline	2.55 ± 0.47	2.49 ± 0.61	0.724
	3 months	0.05 ± 0.12	0.06 ± 0.15	0.894
	6 months	0	0.17 ± 0.39	0.266
	Δ 6 m	2.55 ± 0.47	2.32 ± 0.69	0.759
PI score, n	Baseline	1.12 ± 0.74	1.22 ± 0.70	0.992
	3 months	0.1 ± 0.19	0.2 ± 0.21	0.345
	6 months	0.07 ± 0.13	0.05 ± 0.1	0.996
	Δ 6 m	1.05 ± 0.69	1.17 ±0.7	0.767

Note: PPD, CAL, MM, KM are represented in millimeters. Statistic analysis between the groups by using *t*-tests or Mann–Whitney U test (*p* < 0.05). Abbreviations: PPD—peri-implant probing depth; CAL—clinical attachment level; MT—mucosal thickness; KMW—keratinized mucosa width; mBlI—modified bleeding index score; PI—plaque index score; Δ 6 m—baseline—6 months.

**Table 3:** Radiography outcomes are represented at baseline and six months postoperatively.

		Test Group			Control Group		
		Baseline	6 Months	Δ BG	Baseline	6 Months	Δ BG
V- MB level Mean ± SD	Mesial	3.24 ± 1.44	0.22 ± 0.34	3.02 ± 1.01 *	2.65 ± 1.36	0.68 ± 1.54	1.97 ± 0.43 *
	Distal	5.15 ± 2.38	0.77 ±1.56	4.38 ±1.6 *	3.25 ±1 0.31	0.39 ± 0.42	2.89 ± 0.9 *
	Buccal	3.76 ± 1.7	0.71 ± 0.5	3.05 ± 1.04	3.65 ± 1.32	0.2 ± 0.25	3.45 ± 1.11
	Oral	4.44 ± 2.1	0.29 ± 0.33 *	4.15 ± 1.77 *	2.41 ± 1.14	0.77 ± 1.4 *	1.63 ± 0.4 *
H- MB level Mean ± SD	Mesial	3.81 ± 0.9	0.42 ± 0.92	3.39 ± 0.2	3.1 ± 0.9	0.16 ± 0.32	2.94 ± 0.7
	Distal	3.51 ± 1.24	0.24 ± 1.1	3.27 ± 0.32	2.1 ± 1.12	0.17 ± 0.31	1.93 ± 0.75

Note: V-MB level—vertical marginal bone level; H-MB level—horizontal marginal bone level; Δ BG—bone gained at six months measurements; * Statistically significant difference between the groups by using *t*-tests or Mann–Whitney U test (*p* < 0.05).

## Data Availability

The study design was registered with ClinicalTrials.com numbered NCT05171582.
